# Texture based skin lesion abruptness quantification to detect malignancy

**DOI:** 10.1186/s12859-017-1892-5

**Published:** 2017-12-28

**Authors:** Recep Erol, Mustafa Bayraktar, Sinan Kockara, Sertan Kaya, Tansel Halic

**Affiliations:** 1 Department of Computer Science, UCA, Conway, AR 72034 USA; 2 Bioinformatics, UA Little Rock, Little Rock, AR 72204 USA; 3 HP, San Diego, CA 92127 USA

**Keywords:** Pigmented lesions, Skin lesion, Level set, Contour contraction, Abrupt cutoff

## Abstract

**Background:**

Abruptness of pigment patterns at the periphery of a skin lesion is one of the most important dermoscopic features for detection of malignancy. In current clinical setting, abrupt cutoff of a skin lesion determined by an examination of a dermatologist. This process is subjective, nonquantitative, and error-prone. We present an improved computational model to quantitatively measure abruptness of a skin lesion over our previous method. To achieve this, we quantitatively analyze the texture features of a region within the lesion boundary. This region is bounded by an interior border line of the lesion boundary which is determined using level set propagation (LSP) method. This method provides a fast border contraction without a need for extensive boolean operations. Then, we build feature vectors of homogeneity, standard deviation of pixel values, and mean of the pixel values of the region between the contracted border and the original border. These vectors are then classified using neural networks (NN) and SVM classifiers.

**Results:**

As lower homogeneity indicates sharp cutoffs, suggesting melanoma, we carried out our experiments on two dermoscopy image datasets, which consist of 800 benign and 200 malignant melanoma cases. LSP method helped produce better results than Kaya et al., 2016 study. By using texture homogeneity at the periphery of a lesion border determined by LSP, as a classification results, we obtained 87% f1-score and 78% specificity; that we obtained better results than in the previous study. We also compared the performances of two different NN classifiers and support vector machine classifier. The best results obtained using combination of RGB color spaces with the fully-connected multi-hidden layer NN.

**Conclusions:**

Computational results also show that skin lesion abrupt cutoff is a reliable indicator of malignancy. Results show that computational model of texture homogeneity along the periphery of skin lesion borders based on LSP is an effective way of quantitatively measuring abrupt cutoff of a lesion.

## Background

Melanoma is one of the deadliest and fastest growing cancer types in the world. In the USA annually 3.5 million skin cancers are diagnosed. Skin cancer is rarely fatal except melanoma which is the 6th common cancer type in the USA [1]. Women 25–29 years of age are the most commonly affected group from melanoma. Ultraviolet tanning devices are listed as known and probable human carcinogens along with plutonium and cigarettes by World Health Organization [1]. In 2017, an estimated 87,110 adults were diagnosed with melanoma in the USA and approximately 9730 were fatal [2].

Melanoma is a malignancy of melanocytes. Melanocytes are special cells in skin located in its outer epidermis. Since melanoma develops in epidermis, it can be seen by human eye. Early diagnosis and treatment are critical to prevent harm. When caught early, melanoma can be cured through excision operation. However, high rate of false-negative of malignant melanoma is the main challenge for early treatments [3].

Dermoscopy, a minimal invasive skin imaging technique, is one of the viable methods for detecting melanoma and other pigmented skin proliferations. In the current clinical settings, first step of dermoscopic evaluation is to decide whether the lesion meloanocytic or not. The second step is to find out whether the lesion is benign or malignant. There are commonly accepted protocols to detect malignancy in skin lesions, which are ABCD Rule, 7-point Checklist, Pattern Analysis, Menzies Method, Revised Pattern Analysis, 3-point Checklist, 4-point Checklist, and CASH Algorithm [3, 4].

Celebi et al. [5] extracted shape, color, and texture features and fed these feature vectors to classifier such that they are ranked using feature selection algorithms to determine the optimal subset size. Their approach yielded a specificity of 92.34% and a sensitivity of 93.33% using 564 images. In their seminal work, Dreiseitl et al. [6] analyzed the robustness of artificial neural networks (ANN), logistic regression, k-nearest neighbors, decision trees, and support vector machines (SVMs) on classifying common nevi, dysplastic nevi, and melanoma. They addressed three classification problems; dichotomous problem of separating common nevi from melanoma and dysplastic nevi, and the trichotomous problem of genuinely separating all these classes. They reported that on both cases (dichotomous and trichotomous) logistic regression, ANNs, and SVMs showed the same performance, whereas k-nearest neighbor and decision trees performed worse.

Rubegni et al. [7] extracted texture features, besides color and shape features. Their ANN based approach reached the sensitivity of 96% and specificity 93% on a data set of 558 images containing 217 melanoma cases. Iyatomi et al. [8] proposed an internet-based system which employs a feature vector consists of shape, texture, and color features. They achieved specificity and sensitivity of 86% using 1200 dermoscopy images. Local methods have also been recently applied for skin lesion classification. Situ et al. [9] offered a patch-based algorithm which is to use Bag-of-Features approach. They sampled the region of lesion into 16 × 16 grid and extracted Wavelets and Gabor filters as collecting 23 features in total. They compared two different classifiers which are Naïve Bayes and SVM; the best performance they achieved is 82% specificity on a dataset consists of 100 images with 30 melanoma cases.

A considerable number of systems have been proposed for melanoma detection in the last decade. Some of them aim to mimic the procedure that dermatologists pursue for detecting and extracting dermoscopic features, such as granularities [10], irregular streaks [11], regression structure [11], blotches [12], and blue-white veils [13]. These structures are also used by dermatologists to score the lesion based on seven point-checklist. Leo et al. [14] described a CAD system that mimics the 7 point-checklist procedure.

However, approaches [5, 7, 15, 16] in the literature dominantly pursued pattern recognition in melanoma detection. Majority of these works are inspired by the ABCD rule [17], and they extract the features according to the score table of ABCD protocol. Shape features (e.g. irregularity, aspect ratio and maximum diameter, compactness), which refer to both asymmetry and border, color features in several color channels and texture features (e.g., gray level co-occurrence matrix) [5] are the most common features analyzed when ABCD protocol is used [17]. There are other approaches [15, 18, 19] that used one type of feature for detection of melanoma. Seidenari et al. [15] aim to distinguish atypical nevi and benign nevi using color statistics in the RGB channel, such as mean, variance, and maximum RGB distance. Their approach reached 86% accuracy, additionally they concluded that there is a remarkable difference in distribution of pigments between the populations they studied. Color histograms have been utilized for discriminating melanomas and atypical or benign nevi [18, 19] with specificity little higher than 80%.

## Methods

### Dermoscopic image analysis

**Fig. 1:**
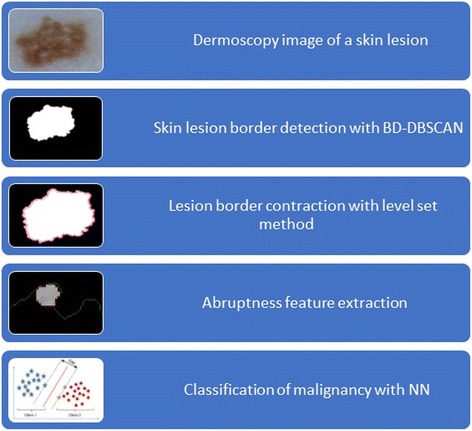
Global work-flow is shown

The dataset for this study is obtained from ISIC 2016: Skin Lesion Analysis Toward Melanoma Detection [20] which has 900 dermoscopic images with 727 benign and 173 malignant lesions, and Edra Interactive Atlas of Dermoscopy [21] which has 73 benign and 27 malignant lesions. The processing steps for this study is given in Fig. 1.

**Fig. 2:**
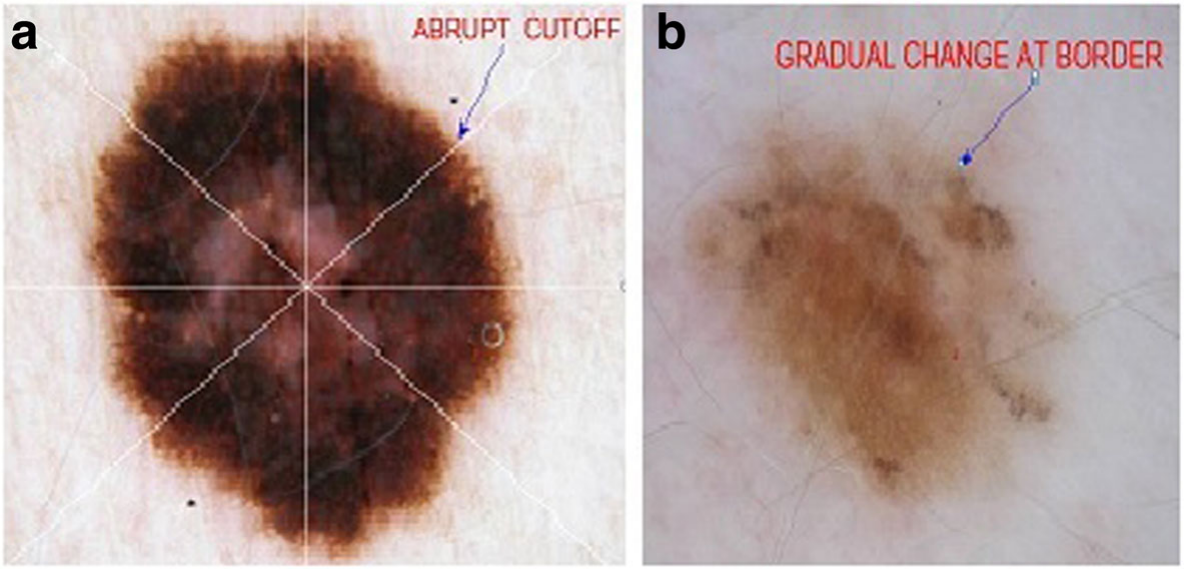
**a** represents a malignant case with abrupt cutoff where the lesion is divided into eight pieces and asterisks indicate abrupt cut off **b** represents a benign case with gradual change at lesion border. In both cases, homogeneity feature is a strong indicator for evaluating the abruptness

In this study, we focus on border abruptness feature of skin lesions. The abrupt cutoff is a commonly accepted clinical indicator of malignancy of a lesion. Assessment of abrupt cutoff in current clinical practice is performed by dividing the lesion into eight virtual pies (see Fig. 2). Dermatologists search abrupt cutoff and assign a score for each of the pie pieces. Since this process is carried out manually, it leads subjective outcomes depending on the experience of the dermatologist examining the lesion. To objectively measure and evaluate abruptness, we first segment the skin lesion using Boundary Driven- Density Based Spatial Clustering Application with Noise (BD-DBSCAN) [22]. Then, we consider the offset of a continuous function of whole lesion border via constant velocity level sets and contract the lesion border using these level sets. Next, we compute texture homogeneity in the designated circular region which resides between actual and contracted lesion border. Kaya et al. [23], was the first work addresses the quantification of abruptness toward melanoma detection. In the current study, we enhance the prior work [1] in two aspects; i-) offering a formal curve offsetting method based on the level set propagation (LSP) which generates better and non-overlapping contracted (inner) border [24], ii-) using NN as a classifier on an extended data set. While first contribution yields us to collect more relevant data during feature extraction, second contribution leads to improve accuracy on the extended dataset and that indicates generalizability of the developed method on greater dataset over Kaya et al. [23] method.

### Boundary detection and boundary contour extraction

**Fig. 3:**
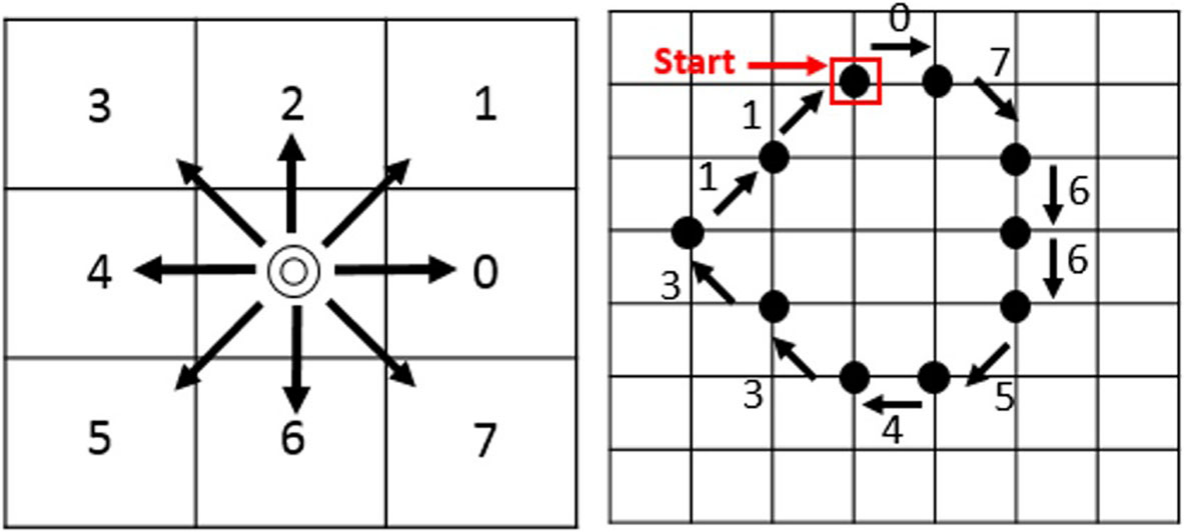
Chain code initialization is shown

To access the region where abrupt cutoff possibly exists, first we need to segment the lesion and extract the lesion border. A novel density based clustering algorithm [22] is used for lesion segmentation. Segmented image is recorded as black and white pixels where black pixels are background and white pixels are foreground (refers to the lesion). To obtain the 2D contour information of the lesion border, we use the chain-code algorithm proposed by Freeman [25]. The chain-code encodes boundary in a binary representation. These encodings refer to 8 possible directions of a neighboring pixel of a starting pixel. These directions range from 0 to 7 in the rectangular-grid. Each number refers to a transition on the direction in between two consecutive points. As can be seen in the rectangular grid given in Fig. 3a and b, direction numbers increase in the counter-clockwise.

**Fig. 4:**
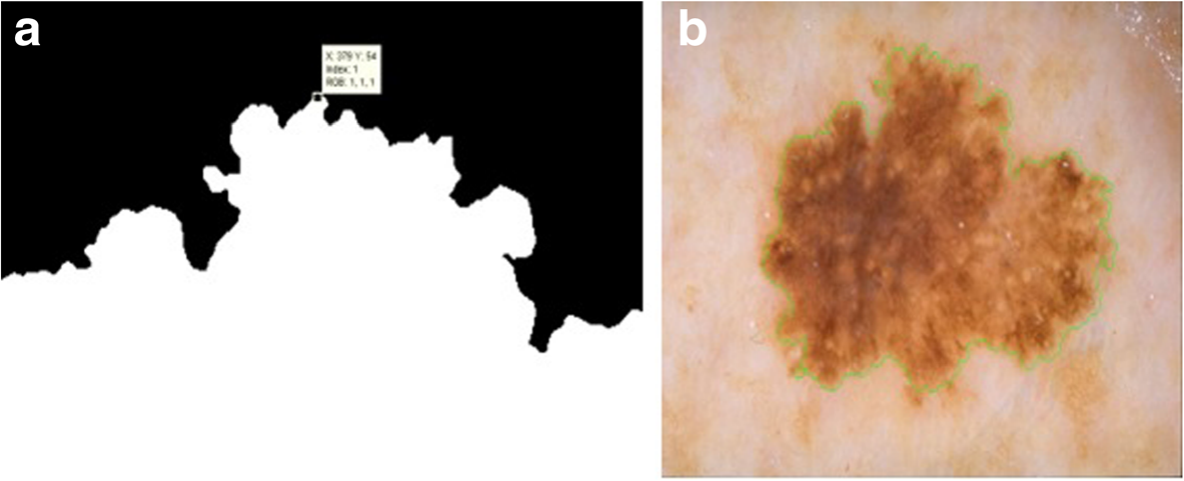
**a** The starting point is shown in (**a**). **b** Lesion boundary is represented in *green*

In chain-code, first among all the pixels belong to foreground, the spatially minimum pixel is selected to start computation. The starting pixel is shown in Fig. 4a with its minimum (X,Y) coordinates. After applying the chain code, the boundary of the lesion is captured as depicted in Fig. 4b (in green).

### LSP for lesion border contraction

**Fig. 5:**
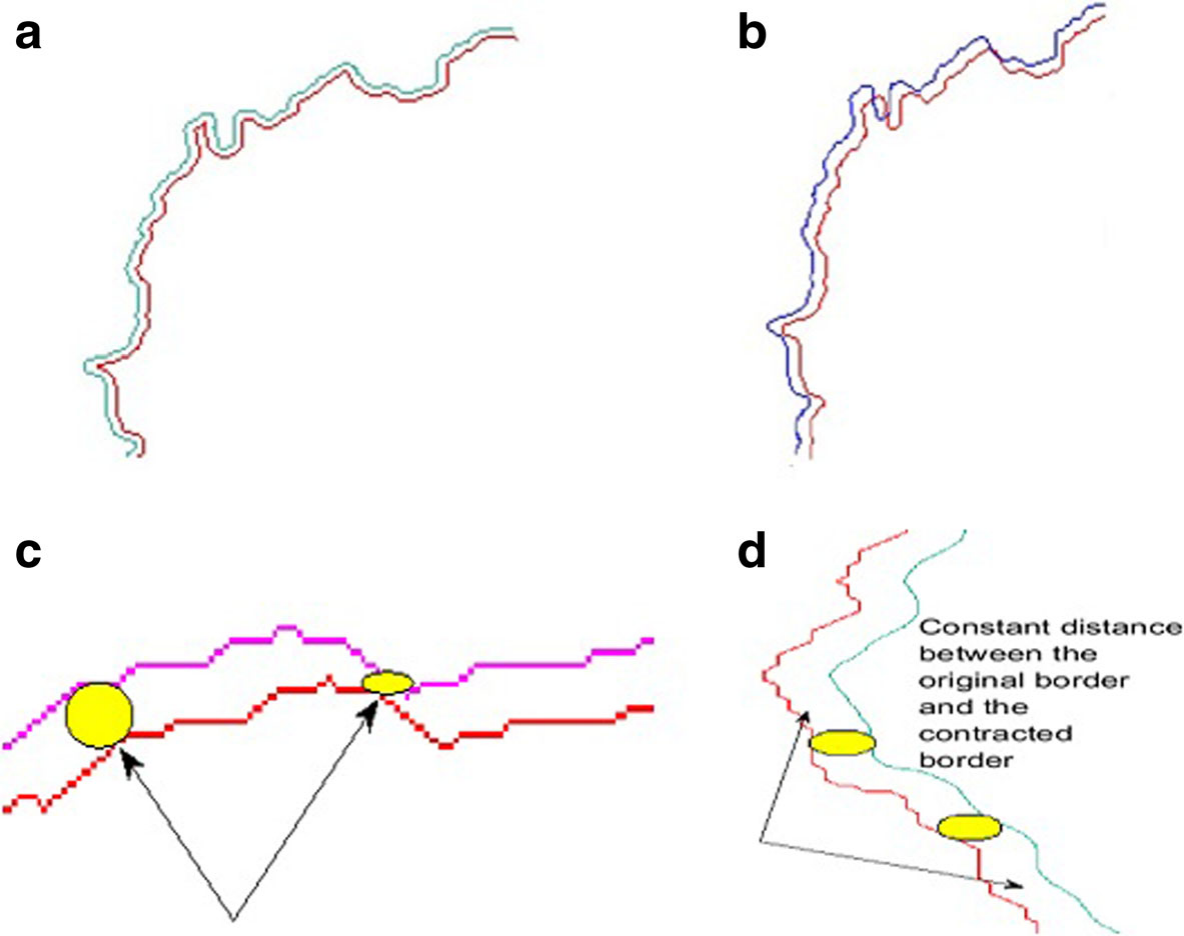
In **a** and **b**, *red* curves represent the contracted border. **a** The curve set shows that the LSP can obtain quantitatively accurate results. **b** The curve set shows the DS still suffers from high curvatures and cannot offer constant distance from the original curve. **c** shows that the DS yields a deficient data collection along the layer where the abruptness is searched. Yellow brushes indicate that not equal amount of territory considered for feature extraction in spanning windows. Note that, these regions are masked using polygon intersection operations prior to feature extraction. **d** shows that the constant velocity LSP imbues equalization of data amount during feature extraction

In our previous study [23], we developed a geometric model for border contraction called dynamic scaling (DS). Interested reader is referred to [23] for details and mathematical foundation for the DS. In this study, we use level set method [24] for border contraction. Previous method of contraction fails to provide equal distance contraction for all the cases especially with very irregular lesion contours, and yields unequal data collection during feature extraction. Whereas, level set based contraction method results in constant proximity between original and contracted border. These can be seen in Fig. 5 a, b, c and d.

Shape contraction algorithms play an important role in computer graphics, computer-aided design, manufacturing, CNC machines. We adopted the method studied in a seminal paper of Kimmel et al. [24]. Following set of formulations give the details of this approach.

In order to formulate shape offsetting/contraction problem, let us parameterize a curve as in the following form.

1 $$ {X}_0(s)={\left[x(s),y(s)\right]}^T $$

where *s* is a curve parameterization factor for curve *X*_0_ . Let us find an offset curve in a closed form, which is expressed as,

2 $$ {X}_L(s)={X}_0(s)-N\left(s,0\right)L $$

Equation [2] formulates a curve leaning “parallel” to *X*_0_(*s*), where *L* is the displacement of the offset curve, and *N*(*s*, 0) represents the unit normal at a *x*_0_(*s*) point and can be written as,

3 $$ N\left(s,0\right)=\frac{1}{\sqrt{x_s^2(s)+{y}_s^2(s)}}{\left[{y}_s(s),{x}_s(s)\ \right]}^T $$

where *N*(*s*, 0) is the normal of the parametric point [*y*_*s*_(*s*), *x*_*s*_(*s*)] on the curve at time 0 (e.g. N(s,0)). For instance, when L is equal to 1, displacement of each iteration will be a single pixel. Let us consider that *X*(*s*, *t*) changes continuously by time (e.g. number of iterations), hence for all *t*, *X*(*s*, *t*) = *X*_0_(*s*) − *tN*(*s*, 0). The term of *tN*(*s*, 0) is negative because we do contraction, it will become positive if expansion is needed. Differential description of this curve evolution becomes as in the following form.

4 $$ \left\{\begin{array}{ccc}\frac{\partial X\left(s,t\right)}{\partial t}& =& -N\left(s,0\right)\\ {}X\left(s,0\right)& =& {X}_0(s)\end{array}\right\} $$

For the first iteration t is equal to 0; thus, curve will remain same, which is represented as *X*(*s*, 0) = *X*_0_(*s*). Eq. 4 suggests that motion of each point on the border (e.g. pixel) will be in inward direction (due to the contraction) of the normal as given in Eq. 5.

5 $$ N\left(s,t\right)={\left[{y}_s(s),{x}_s(s)\right]}^T\frac{1}{\sqrt{x_s^2(s)+{y}_s^2(s)}} $$

**Fig. 6:**
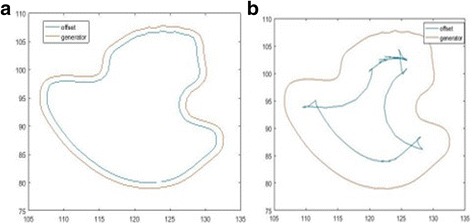
**a** Without entropy condition stability can be preserved if contraction distance is less than the curvature of an arbitrary 2d curve; **b** Cusps emerge when contraction distance is greater than the curvature. Shocks and cusps can be avoided adopting entropy condition

Here constant 1 in numerator of the fraction refers to the velocity during the curve propagation at time t. For faster contraction, velocity or time step may be increased. Eq. 5 yields time *t* dependent shape offsets for *t* > 0. Figure 6b illustrates deficiency of selecting bigger time step or higher velocity values where displacement factor L becomes larger than the curvature. Thus, it results in loss of silhouette of actual curvature. To overcome these possible problems (also called singularities or shocks), we employ a more stable technique based on flame-propagation model given in [24].

Shocks occur when normal of original curve collide or cross itself, in other words when the curvature of *X*_0_ becomes singular. To address this constraint, Huygens applies “entropy condition” on the evolving curve. Osher and Sethian [26] offered an efficient and numerically stable wave front propagation for the curves in the plane to overcome self collision problem. Osher et al. [26] applied Huygens principle, which is also known for adhering entropy condition, proposing a solution for Eq. 5 such that *X*(*s*, *t*) at time *t* is the approximation of the whole class of disks of time *t* centered along the original curve *X*_0_(*s*). We adopted Osher’s method [25] with entropy condition to contract curve to obtain more accurate results as given in Eq. 6 while eliminating self collision problem. Due to the front dependency of the parameters *s* and *t*, a Langrangian numerical-propagation scheme may be used to approximate the curve propagation as in the following form.

6 $$ \left\{\begin{array}{ccc}\frac{\partial \boldsymbol{x}\left(s,t\right)}{\partial t}& =& \frac{y_s\left(s,t\right)}{\sqrt{x_s^2\left(s,t\right)+{y}_s^2\left(s,t\right)}}\\ {}\frac{\partial \boldsymbol{y}\left(s,t\right)}{\partial t}& =& \frac{x_s\left(s,t\right)}{\sqrt{x_s^2\left(s,t\right)+{y}_s^2\left(s,t\right)}}\end{array}\right\} $$

Numerical-propagation scheme takes central derivatives of *x* and *y* in location *s*, and forward-derivative in time *t*. However, Langrangian based numerical propagation of a curve given in Eq. 6 is unstable and suffers from aforementioned topological problems, i.e. shocks, self-intersections (a.k.a. self collision). To maintain stability and address topological problems, instead of Langrangian numerical propogation, we use the ‘Eulerian formulation’.

### Eulerian formulation

Eulerian approach implements the entropy condition inherently by a recursive procedure. Let us define a function *ϕ*(*x*, *y*, *t*) and initialize it as *ϕ*(*x*, *y*, *t*) = 0 that results in a closed curve ***X***(s, 0). *ϕ* is strictly negative inside and outside of the level set *ϕ*(*x*, *y*, 0) = 0. The rationale behind this approach is to search for the surface evolution of *ϕ*(*x*, *y*, *t*), hence level sets *ϕ*(*x*, *y*, *t*) = 0 yield the propagated curves ***X***(s, t) preserving the entropy condition. Let us consider *ϕ*(*x*, *y*, *t*) = 0 along ***X***(s, t), therefore chain rule yields to:

7 $$ {\displaystyle \begin{array}{l}\frac{\partial \boldsymbol{x}\left(s,t\right)}{\partial t}+\frac{\partial \phi \left(\boldsymbol{x}\left(s,t\right),y\left(s,t\right),t\right){x}_t}{\partial x}+\frac{\partial \phi \left(\boldsymbol{x}\left(s,t\right),y\left(s,t\right),t\right){y}_t}{\partial y}=0\\ {}\mathrm{or}\\ {}{\phi}_t+\nabla {X}_t\left(s,t\right)=0\end{array}} $$

where,

8 $$ \nabla \varnothing =\left[\frac{\partial \varnothing }{\partial x},\frac{\partial \varnothing }{\partial y}\right] $$

represents the gradient of ∅(*x*, *y*, *t*) for point (*x*, *y*) at time *t*. Following equation is to derive a connection with the scalar velocity of each point on the curve and its normal direction:

9 $$ v=\boldsymbol{N}\left(\mathrm{s},\mathrm{t}\right).{\boldsymbol{X}}_{\boldsymbol{t}}\left(\mathrm{s},\mathrm{t}\right) $$

Here, we constrain *v* = 1 to have 1 pixel displacement for a single time step. Since the gradient is always normal to the curve, it will be equal to zero as ∅(*x*, *y*, *t*) = 0 ; therefore,

10 $$ N\left(s,t\right)=-\frac{\nabla \varnothing }{\left\Vert \nabla \varnothing \right\Vert } $$

where negativity indicates that the direction of propagation is inward (contraction); thus,

11 $$ v=\boldsymbol{N}.{\boldsymbol{X}}_{\boldsymbol{t}}=-\frac{\nabla \varnothing }{\left\Vert \nabla \varnothing \right\Vert }{\boldsymbol{X}}_{\boldsymbol{t}}=1 $$

Embedding Eq. 12 into Eq. 8 results in the surface evolution as in the following form.

12 $$ {\varnothing}_t-\left\Vert \nabla \varnothing \right\Vert =0 $$

Solution for partial differential equation given in Eq. 13 can be carried out considering Hamilton-Jacobi Equations and gradient descent. Algorithm 1 (see Algorithm 1) summarizes steps for the LSP to generate contracted border. Figure 5 illustrates results of contracted borders generated from the DS method and the LSP. As seen from Fig. 5, the LSP eliminates problems such as shocks and self-intersections whereas these problems exist with DS. Interested readers are referred to [24] for detailed mathematical derivations of the LSP.
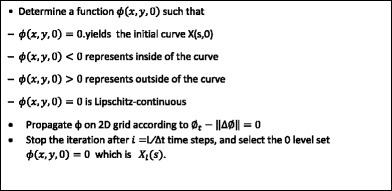


After contracted border is found with LSP method, we calculate texture homogeneity between lesion border and contracted border with various radii sizes.

### Feature extraction

We obtain three different statistical measures which are mean, standard deviation, and a texture descriptor Gray Level Co-occurrence Matrix (GLCM) as a homogeneity indicator [27]. GLCM is a statistical method that is to analyze texture characteristics of an image which relies on the spatial dependency of pixels. Mathematical representation of GCLM is given below,

13 $$ {C}_{\varDelta x\varDelta y}\left(i,j\right)={\sum}_{p=1}^n{\sum}_{q=1}^m\left\{\begin{array}{cc}1,& if\ I\left(r,t\right) and\ I\left(p+\varDelta x,q+\varDelta y\right)=u\\ {}0,& otherwise\end{array}\right\} $$

where *I* is an image with *nxm* size, *C* is the co-occurrence of intensity value *u*, (∆*x*, ∆*y*) is an offset parameter, and lastly *r* and *t* are the spatial coordinates in the image *I(r,t)*. Note that, offset parameters make the co-occurrence matrix variant to rotation.

**Fig. 7:**
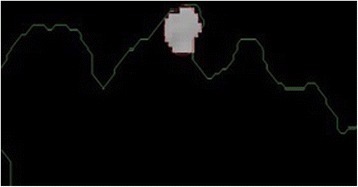
Homogeneity extraction from the highlighted region along the lesion boundary

Various statistical features (texture related) can be obtained by deploying the GCLM matrix, such as contrast, correlation, energy, and homogeneity. Here, we focus on homogeneity which measures the similarity of grey level distribution on the image. Hence, the homogeneity can be expressed as in the form given in Eq. 14 where m and n respectively represent the number of image pixels in the vertical and horizontal directions. Figure 7 illustrates a sample region where homogeneity feature is extracted.

14 $$ {\sum}_{i=1}^m{\sum}_{j=1}^n\frac{GLCM\left(i,j\right)}{1+\left|i-j\right|} $$

After border contraction using the LSP and extracting homogeneity features in GLCM, next step is to analyze generated data.

## Data analysis and results

After feature extraction step, we categorized dataset according to thickness of layer they are collected from. As mentioned in the abstract, we selected 5, 7, 10, and 15 as the radius of circles between border and contracted border, and the layer is generated by enveloping these circles. In each overlapping circles (patches), we compute the “mean-homogeneity”, “min-homogeneity”, “mean- color value average”, “minimum color value average”, “mean color value standard deviation”, and “minimum color standard deviation”. We performed the experiments on two different color spaces which are RGB and HSV and fed them as input to the NN architectures and SVM.

Dataset provides dermoscopy images which are labeled either as malignant or benign. We are measuring abruptness of lesion along the periphery of the lesion border using homogeneity features to conduct binary classification. Here, we argue that Multi-layer Perceptron-based Neural Networks (MPNN) have ability to compete with SVM, when it is combined with softmax regression.

The hidden layer system can include multi-layers within separate instances better and converge the values efficiently. A careful design of a NN is required for obtaining higher accuracy rates in classification. There are some parameters that the user needs to tune [28] for the best accuracy, such as input layer selection, weights, the number of hidden layers, the number of nodes on each hidden layer, activation function, learning rate, the number of iterations, and cost minimization function. We train our NN with a pair of input feature values and output malignancy values. In our study, in order to solve the malignancy problem of the dataset, we choose two NN architectures; multi-layer perceptron and the fully-connected multi-hidden layer NN.

**Fig. 8:**
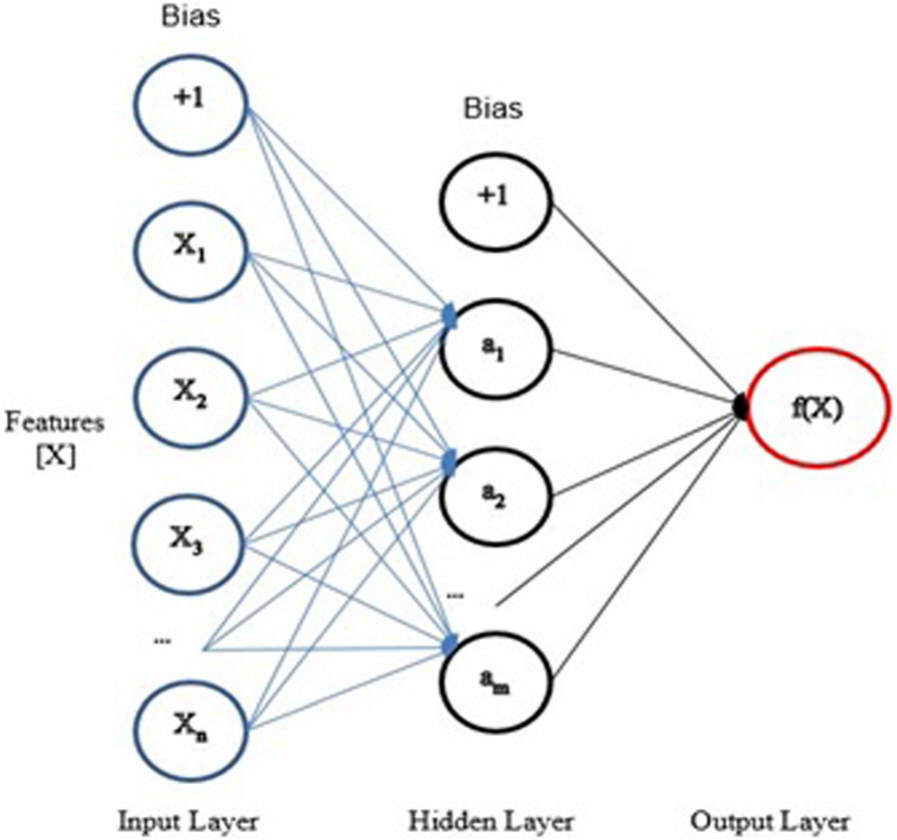
Multi Layer Perceptron with a single hidden layer NN architecture

The first architecture we used is NN models multi-layer perceptron binary classification [29]. In this architecture, we used standard single layer NN which consists of input layer, single hidden layer, and output layer. Figure 8 schemes the architecture. In the input layer of this NN, we used three different inputs which are RGB channels, HSV channels, and RGB-HSV combined channels. The number of features for RGB, HSV, and RGB-HSV channels are 18, 18, and 36, respectively. In the hidden layer, we used the same size as they are in the input layer. In the output layer, two classes’ values that are “benign” and “malignant” are converted to “0” and “1”, respectively. In the running process of this NN, each epoch has one feed forward and one back propagation. After empirical trials, execution continued at most 1000 iterations or execution stopped when the learning rate between each epoch is less than or equal to 0.001. The rectified linear unit (ReLU) is chosen as the activation function for this NN.

**Fig. 9:**
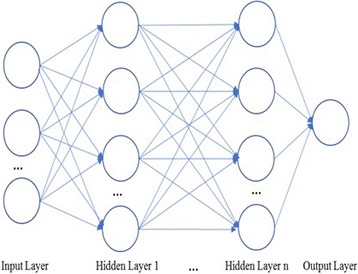
Fully-connected multi-hidden layer NN architecture

The architecture of the second NN is fully-connected multi-hidden NN network. Figure 9 illustrates the architecture of its design such that in this NN, the input layer is the same with the previous NN. The hidden layer is designed with the Softmax regression [30]. In the output layer, benign and malignant values are converted to one-hot encodings which are [1 0] or [0 1], respectively. The implementation of this design is done using TensorFlow NN library [31].

We obtained results of two different abrupt cutoff feature extraction methods; Kaya et al. [23] and our LSP based method using the two NN architectures introduced above with same parameters. Optimum results are obtained from the features collected when radius is 10 and on RGB channel. NNs are highly sensitive to hyper-parameter changes, we applied tunings to get optimum results. We empirically determined the iteration numbers as 600, 750, and 1000 without constraining a stoppage criterion. Then, we added the learning rate of 0.0001 to exit the iteration between two consecutive epochs. We applied 10-fold cross-validation to split the data into training and test sets. Since NNs generate random weights between the layers at each time, we run the algorithms 10 times. Consequently, all evaluation metrics are the average of the all results generated in these experiments. Notably, to maintain consistency we used same dataset to test our NN designs.

We run both NN methods and SVM on the same set of image data however different feature vectors based on the different feature extraction methods used (the LSM and the DS). Table 1 shows the results obtained from the multi-layer perceptron NN, fully connected multi- hidden layer NN, and SVM classifiers which are fed by features extracted using both the LSP and the DS methods. Table 2 is shows the parameters of the all classifiers used in the experiments. The highest f1-score, 87% with 78% specificity, is obtained using fully connected multi-hidden layer NN in the RGB combination with the radius 10.

**Table 1:** LSP vs. DS based texture homogeneity feature extraction and classification of lesions with various classifiers: multi-layer perceptron, fully connected multi-hidden layer NN, and SVM. 10-fold cross-validation is used. Results listed here are means of 10 random executions

Feature Extraction- Classification	Precision	Recall	Sensitivity	F1-Score
LSP-Multilayer Perceptron NN	0.82	0.81	0.75	0.8
DS-Multi Layer Perceptron NN	0.77	0.76	0.56	0.74
LSP-SVM	0.69	0.64	0.66	0.66
DS-SVM	0.62	0.61	0.61	0.61
LSP-Fully-connected multi-layer NN	0.86	0.87	0.78	0.87
DS-Fully-connected multi-hidden layer NN	0.76	0.75	0.61	0.75

**Table 2:** The parameters of the NN (the multi layer perceptron and the fully-connected multi-hidden layer NN) classifiers and SVM

Parameters	NN	Parameters	SVM
Learning Rate	0.001	Kernel Function	Polynomial
Number of iteration	1000	Polynomial Order	3
Number of run	20	Kernel Scale	auto
Number of hidden layer	1	Box constraint	inf
Number of hidden layer node	4	Standardize	TRUE
Number of hidden layers (If multilayer NN is used)	4	Outlier Fraction	0.05

## Conclusions

An improved automated measurement of abrupt cutoff for skin lesions is presented. LSP over dynamic scaling to do lesion border contraction is introduced. Computational results showed that skin lesion abrupt cutoff is a worthy indicator of malignancy. Results show that computational model of texture homogeneity along the periphery of skin lesion borders is an effective tool to quantitatively measure abrupt cutoff of a lesion. A multi-layer perceptron and a fully connected multi-hidden layer NN, and SVM classifiers are used. We obtained 87% f1-score and 78% specificity for correctly classifying lesions with the fully-connected multi-hidden layer NN classifier and LSP based border contraction method.
